# Association Between Maternal Lifestyle and Risk of Metabolic Syndrome in Offspring—A Cross-Sectional Study From China

**DOI:** 10.3389/fendo.2020.552054

**Published:** 2020-12-14

**Authors:** Yanhui Li, Zhaogeng Yang, Xijie Wang, Di Gao, Zhiyong Zou, Bin Dong, Jun Ma, Luke Arnold

**Affiliations:** ^1^ Institute of Child and Adolescent Health, School of Public Health, Peking University, Beijing, China; ^2^ Department of Commissioning, South Western Sydney Primary Health Network, Campbelltown, Australia

**Keywords:** metabolic syndrome, adolescent, lifestyle, maternal behavior, offspring

## Abstract

**Background:**

The prevalence of MS among children and adolescents continues to rise, which has become an escalating serious health issue worldwide. It had been reported that maternal current lifestyle had a strong independent correlation with offspring health. However, it is not clear whether comprehensive lifestyle of mother has an impact on the MS risk in offspring and the role of offspring’s lifestyle in it.

**Methods and Results:**

We included 4,837 mother-child pairs from a multi-centered cross-sectional study conducted in China. The information of maternal lifestyle was obtained by self-reported questionnaire, and metabolic syndrome (MS) in offspring was determined by anthropometric measurements and blood tests. Logistic regression models were employed to evaluate the association between maternal lifestyle and risk of MS in offspring. We found maternal healthy lifestyle was independently associated with lower risk of offspring MS, and the risk of MS in offspring decreased with the increased number of maternal ideal lifestyle factors. Although adolescents’ lifestyle did not fully explain the relationship between maternal lifestyle and risk of offspring MS, compared with those had less ideal lifestyle factors in both mothers and offspring, the risk of offspring MS was lower in those had more ideal lifestyle factors in both mothers and adolescents.

**Conclusions:**

Healthy lifestyle in mothers was associated with a lower risk of MS in offspring, which was independent of offspring’s lifestyle. These findings support mother-based lifestyle intervention could be an effective strategy to reduce the MS risk in adolescents.

## Introduction

Metabolic syndrome (MS) is a complex multifactorial disorder characterized by a combination of central obesity, hypertension, high triglycerides, low high-density lipoprotein, and high blood glucose. It has been associated with an increased risk for type 2 diabetes, cardiovascular disease, and all-cause mortality (1, 2). Metabolic syndrome usually was described in adults though, its risk factors started at childhood and adolescence. The prevalence of MS among children and adolescents ranged from 0.9 to 11.4% depending on various definition criteria (3). With the increasing prevalence of obesity among children and adolescents, the incidence of MS continues to rise, which has become an escalating serious health issue worldwide (4, 5). Therefore, the identification of modifiable factors during childhood and adolescence could be the effective intervention strategy for reducing MS risk.

Maternal lifestyle has been reported that it was closely related with offspring’s behavior and cardiometabolic health (6–8). Large population-based study and prospective cohort study both showed that maternal current lifestyle, such as body weight, smoking, and alcohol drinking, had a strong independent correlation with offspring weight status and blood pressure (9–12). A recent study considered multiple lifestyle factors as a whole found that mothers with an overall healthy lifestyle had a greater protection on adolescents’ obesity (13). However, it is not clear whether comprehensive lifestyle of mother has an independent impact on the MS risk in offspring when the role of offspring’s lifestyle was excluded.

Therefore, based on national multi-center data, we attempted to explore the independent relationship between maternal lifestyle and the risk of MS in offspring aged 10–18 years with the aim of providing evidence for interventions to reduce MS risk and improve cardio-metabolic health in adolescents. In this study, we hypothesized that maternal current healthy lifestyle was related to lower risk of offspring MS.

## Materials and Methods

### Study Population

Data were obtained from a cross-sectional study conducted in Chinese children and adolescents from seven provinces in 2013. Details about the study design had been described previously (14). In brief, about 12 to 16 schools were enrolled in each region. Two classes were randomly selected in each grade from each school, and all students aged 10–18 years and their mothers in selected classes were invited to participate in this project. Randomization was performed by a staff member who was not involved in this study.

**Figure 1:**
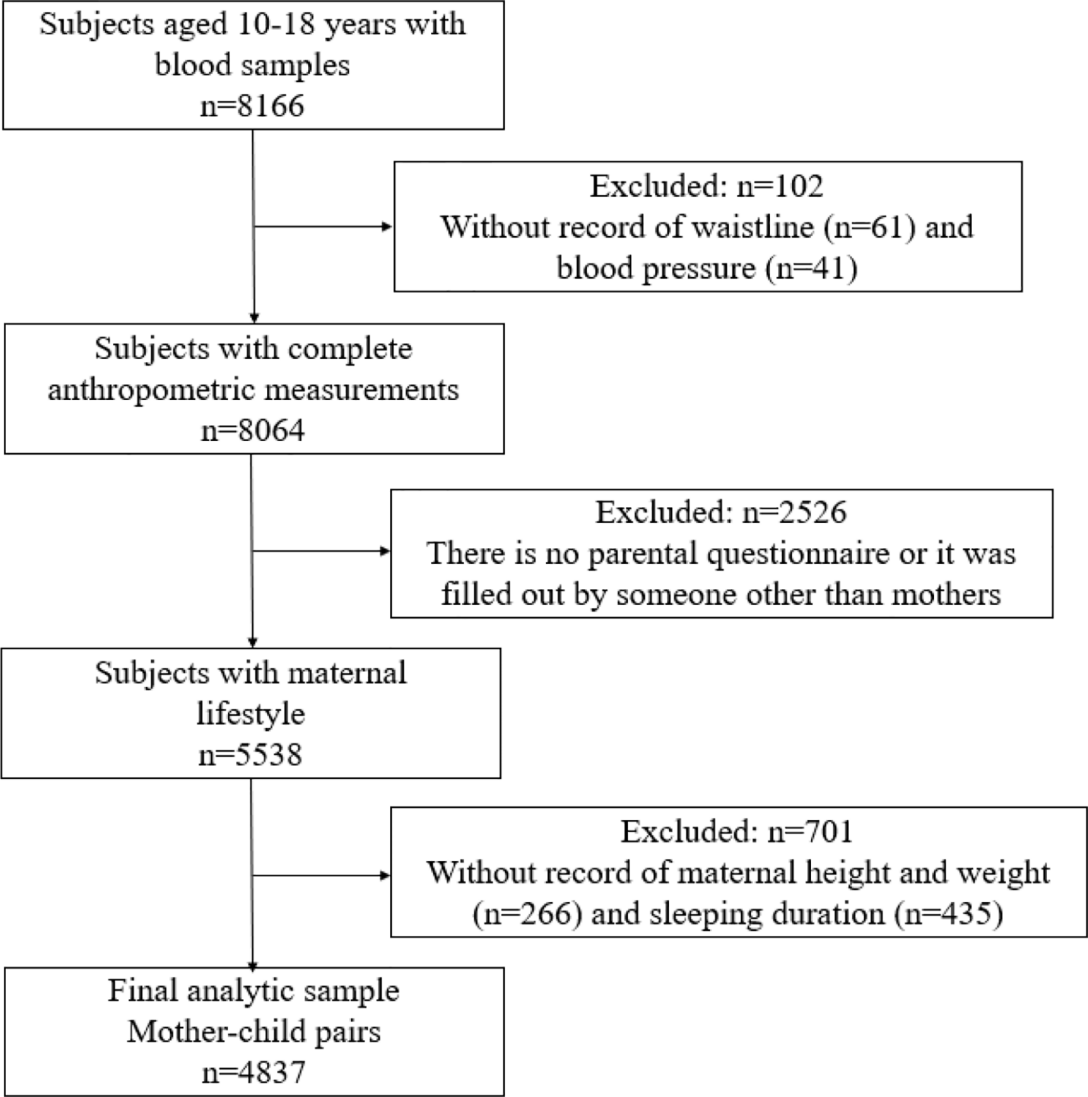
The selection process of the participants.

**Figure 1** shows the selection process of the participants. Of the 5,538 adolescents aged between 10 and 18 years whose blood sample, as well as their maternal questionnaires, were available, 701 participants with missing record on maternal height, weight, and sleeping were excluded. Finally, a total of 4,837 mother-child pairs were enrolled in the study. All participants and their parents have signed informed consents, and this study adheres to the Declaration of Helsinki for ethical standards and has been approved by the Ethical Committee of the Peking University (Number: IRB0000105213034).

### Data Collection and Measurements

All participants underwent anthropometric evaluation in a uniform manner. Height was measured by portable stadiometer (model TZG, China) with an accuracy of 0.1 cm. Weight was measured to the nearest 0.1 kg without shoes and in light clothing by Lever type weight scale (model RGT-140, China). BMI was computed by dividing weight (kg) by height squared (m^2^). Waist circumference (WC) was measured to the nearest mm and located at the 1 cm above umbilicus by steel tape (MT05 by AccuFitness, USA). Blood pressure was measured from the right arm of participants using mercury sphygmomanometer (model XJ11D, China) and stethophone (model TZ-1, China) after at least 5 min of rest. An average of two blood pressure measurements at a single visit was calculated for each participant.

Adolescents’ and parental demographic characteristics, lifestyle, adolescents’ early life factors (including delivery way, feeding type, and birth weight), and family history of diseases were collected using self-filled questionnaires. Lifestyle included cigarette smoking (yes/no), alcohol consumption (yes/no), sleep duration, physical activity, and dietary behavior. Physical activity information was collected by World Health Organization Global Physical Activity Questionnaire, and the frequency and duration of moderate and vigorous intensity activity over the past 7 days was recorded. The average physical activity time per week was calculated as: weekly physical activity time = frequency (days per week) × duration (hours per time). Dietary information, including the intake of vegetables, fruits, sugar-sweetened beverages (SSBs), and meat, was collected by questionnaire and the average daily intake of vegetables and fruits was calculated as: daily intake = (frequency (days per week) × daily servings)/7, in addition, average weekly intake of sugar-sweetened beverages and meat was calculated as: weekly intake = frequency (days per week) × daily servings. One serving of vegetable or fruit is approximately 120 g, one serving of SSBs is approximately 250 ml, and one serving of meat is approximately 100 g.

After overnight fasting for 8 to 12 h, 5 ml venous blood samples were collected for each adolescent. Blood tests were performed by the autoanalyzer (Hitachi 7080, Japan). The fasting blood glucose (FBG), triglycerides (TG), and high-density lipoprotein (HDL-C) tested by glucose oxidase method, enzymatic method, and clearance method, respectively. All analyses were carried out by a qualified biomedical analyses laboratory (14).

### Definitions of MS and Healthy Lifestyle Factors

Metabolic syndrome in offspring was defined according to the criteria of the ATP III modified for age (15). Those who meet three of the five abnormalities, including abdominal obesity, elevated blood pressure, high triglycerides, low HDL-C, and high glucose level, were diagnosed as being MS. According to the definition of MS in offspring, abdominal obesity here referred to the waist circumference ≥90th percentile for sex and age. Elevated blood pressure referred to systolic blood pressure and/or diastolic blood pressure ≥90th percentile for sex, age, and height. While those of TG ≥1.24 mmol/L were considered as having high triglycerides, those with HDL-C ≤1.03 mmol/L were classified as having low HDL-C, and adolescents with fasting blood glucose ≥6.1 mmol/L was defined as having high blood glucose. In this study, the reference values for waist circumference and blood pressure are based on the Chinese population (16, 17).

The definition of healthy lifestyle in mothers was shown in **Table 1**. Six factors, including cigarette smoking, BMI, physical activity, dietary behavior, sleep duration, and alcohol consumption, were considered to define maternal healthy lifestyle. These factors were selected based on the evidence for their association with MS and recommendations for MS prevention and management (18, 19). For each lifestyle factor, meeting the threshold was judged as ideal, otherwise it was considered as unideal.

**Table 1:** Definition of maternal healthy lifestyle factors.

Lifestyle factor	Definition of healthy lifestyle factor
Cigarette smoking	No cigarette smoking during the past week
Body mass index	<25 kg/m^2^
Physical activity	≥150 min/week moderate intensity or ≥75 min/week vigorous intensity or combination
Dietary behavior	Satisfy 3–4 components ◆ Vegetables: ≥4 servings per day ◆ Fruits: ≥3 servings per day ◆ Sugar-sweetened beverages: ≤4.0 servings per week ◆ Total meat consumption: ≤3 servings per week
Sleep duration	7~9 h/day
Alcohol consumption	No alcohol consumption during the past week

The threshold criteria for maternal ideal smoking status, BMI, and physical activity was consistent with the goal for cardiovascular health defined by The American Heart Association (20). Ideal dietary behavior was identified based on four components, including intake of vegetables, fruits, sugar-sweetened beverages, and meat. The threshold of sugar-sweetened beverages consumption was suggested by The American Heart Association (20), while the threshold of vegetables, fruits, and meat consumption were recommended by Dietary Guidelines for Chinese Residents (21). Mothers met three to four dietary components were defined as having ideal dietary behavior. According to the recommendations of National Sleep Foundation, ideal sleep duration was defined as 7–9 h per day (22). For alcohol consumption, the ideal status was defined as no alcohol consumption currently (23).

In offspring, six factors, including no smoking, no alcohol consumption, keeping a healthy body weight (neither wasting nor overweight/obesity) (24), having sufficient sleep duration (25), engaging in regular moderate or vigorous physical activity (≥1 h per day), and eating a healthy diet (met three of four factors of healthy vegetables, fruits, sugar-sweetened beverages and meat consumption) (26), were considered to define healthy lifestyle.

To evaluate the association between maternal comprehensive healthy lifestyle and risk of offspring MS, the number of maternal ideal lifestyle factor were categorized into four groups (0–2, 3, 4, and 5–6). When analyzing the relationship between the maternal and offspring combined lifestyle and MS risk of offspring, maternal and offspring lifestyle were categorized into low-risk and high-risk groups, respectively, based on the number of ideal lifestyle factors. Those who adhered to 4–6 ideal lifestyle factors were considered as low-risk group, while those who met 0–3 ideal lifestyle factors were regarded as high-risk group.

### Statistical Analysis

All analysis was performed with SPSS 24.0 (IBM SPSS Statistics 24.0, USA). Continuous variables were expressed by mean values and standard deviations, and categorical variables were expressed by numbers and percentages. Logistic regression models were employed to evaluate the association between maternal lifestyle and the risk of MS in offspring. Both odds ratios (OR) and 95% confidence intervals (CI) were calculated. The number of maternal ideal lifestyle factor was included in the regression analysis as a continuous variable for trend test. In addition, the association between combined lifestyle of mothers and offspring with the MS risk in offspring was analyzed using logistic regression model. All statistical tests were two sided and were considered statistically significant at *P* < 0.05.

## Results

The characteristics of 4,837 mother-offspring pairs in this study are shown in **Table 2**. Only 1.1% of mothers had their all six factors ideal. More than 80% of mothers had an ideal BMI, cigarette smoking, and alcohol consumption, but the prevalence of ideal dietary behavior was quite low (3.8%). About 8.3% (n = 403) of adolescents were classified as having MS.

**Table 2:** Characteristics of mothers and offspring.

Characteristics		Value
**Maternal characteristics**		
Age, year		39.7 ± 4.5
BMI, kg/m^2^		22.5 ± 2.9
Education level, %		
Less than high school		2,246 (46.7%)
High school diploma or equivalent		1,985 (41.3%)
Bachelor degree or above		581 (12.1%)
Ideal BMI, %		3,994 (82.6%)
Ideal cigarette smoking, %		4,789 (99.0%)
Ideal physical activity status, %		2,359 (48.8%)
Ideal dietary behavior, %		183 (3.8%)
Ideal sleep duration, %		3,174 (65.6%)
Ideal alcohol consumption, %		4,767 (98.6%)
Number of ideal lifestyle factors, %		
1		7 (0.1%)
2		171 (3.5%)
3		1,108 (22.9%)
4		2,213 (45.8%)
5		1,287 (26.6%)
6		51 (1.1%)
**Offspring characteristics**		
Age, year		13.2 ± 2.2
Girl, %		2,588 (53.5%)
Urban, %		2,476 (51.2%)
Natural labor, %		2,982 (62.6%)
Breastfeeding, %		4,143 (86.7%)
Birth weight, g		3,305.5 ± 495.6
BMI, kg/m^2^		20.1 ± 3.9
Systolic blood pressure, mmHg		107.9 ± 11.6
Diastolic blood pressure, mmHg		68.2 ± 8.7
Triglycerides, mmol/L		1.0 ± 0.5
High density lipoprotein, mmol/L		1.3 ± 0.3
Fasting blood glucose, mmol/L		4.8 ± 0.7
Physical activity ≥1h, %		2,668 (62.4%)
Abdominal obesity, %		1,231 (25.4%)
Elevated blood pressure, %		1,203 (24.9%)
High triglycerides, %		1,058 (21.9%)
Low high density lipoprotein, %		797 (16.5%)
High blood glucose, %		16 (0.3%)
Metabolic syndrome, %		403 (8.3%)

BMI, body mass index. Continuous variables were expressed by mean values ± standard deviations, and categorical variables were expressed by frequency values (percentages, %).

The ideal status of maternal lifestyle factors was judged from the definitions in **Table 1**.

As shown in **Table 3**, maternal lifestyle was related with offspring metabolic risk, and the increased number of maternal ideal lifestyle factors was associated with decreased risk of MS in their offspring (*P* for trend = 0.001). The prevalence of MS dropped from 17.4% in adolescents whose mothers had 0–2 ideal factors to 7.4% in those whose mothers had 5–6 ideal factors (*P* for trend < 0.001). The corresponding MS risk in offspring was 51.8% lower in those whose mothers met 5–6 ideal lifestyle factors compared with those whose mother had 0–2 ideal lifestyle factors after adjustment (OR = 0.482, 95%*CI* = 0.287~0.811).

**Table 3:** Associations between maternal lifestyles and risk of offspring MS.

Number of ideal lifestyle factor	N (%) of offspring MS	*χ^2^*	*P*	Risk of offspring MS, OR (95% CI)^†^		
				Model 1	Model 2	Model 3
0–2	31 (17.4%)	21.431	<0.001	1 (reference)	1 (reference)	1 (reference)
3	97 (8.8%)			0.455 (0.293, 0.706)^*^	0.545 (0.325, 0.913)^*^	0.557 (0.314, 0.989)^*^
4	176 (8.0%)			0.410 (0.270, 0.622)^*^	0.476 (0.289, 0.784)^*^	0.450 (0.258, 0.785)^*^
5–6	99 (7.4%)			0.379 (0.244, 0.587)^*^	0.482 (0.287, 0.811)^*^	0.449 (0.251, 0.802)^*^
*P* for trend	<0.001			0.001	0.046	0.023

^*^P < 0.05.

^†^Model 1 did not adjust for any factors.

Model 2 adjusted for region, adolescents’ age and sex, delivery way, feeding type, birth weight, parental education level, parental age, family history of diseases, paternal BMI, paternal cigarette smoking, and paternal alcohol consumption.

Model 3 additionally adjusted for adolescents’ cigarette smoking, adolescents’ alcohol consumption, adolescents’ sleep duration, adolescents’ physical activity, and adolescents’ consumption of vegetables, fruits, sugar-sweetened beverages, and meat.

To explore whether the influence of maternal lifestyle on offspring MS could be explained by the lifestyle of offspring, the model was further adjusted for adolescents’ lifestyle, including cigarette smoking, alcohol consumption, sleep duration, physical activity, and dietary behavior. This adjustment did not change the relationship (**Table 3**, model 3), and the MS risk was 55.1% lower in adolescents whose mother adhered to 5-6 ideal lifestyle factors compared with those whose mothers met 0-2 ideal lifestyle factors (OR = 0.449, 95% *CI* = 0.251~0.802). In the stratified analysis, similar association was detected, though significant association was only found in girls and those lived in urban area (**Table 4**).

**Table 4:** Associations between maternal lifestyle and the risk of offspring MS, stratified by region and gender.

Number of ideal lifestyle factor	Risk of offspring MS, OR (95% CI)^†^			
	Urban	Rural	Boys	Girls
0–2	1 (reference)	1 (reference)	1 (reference)	1 (reference)
3	0.378 (0.165, 0.866)^*^	0.724 (0.312, 1.681)	0.623 (0.242, 1.606)	0.496 (0.233, 1.057)
4	0.352 (0.159, 0.778)^*^	0.552 (0.242, 1.257)	0.512 (0.204, 1.285)	0.418 (0.202, 0.865)^*^
5–6	0.300 (0.130, 0.694)^*^	0.591 (0.253, 1.382)	0.552 (0.213, 1.430)	0.372 (0.173, 0.799)^*^
*P* for trend	0.041	0.218	0.359	0.032

^*^P < 0.05.

^†^In the regression model, we adjusted for region, adolescents’ age and sex, delivery way, feeding type, birth weight, parental education level, parental age, family history of diseases, paternal BMI, paternal cigarette smoking, paternal alcohol consumption, adolescents’ cigarette smoking, adolescents’ alcohol consumption, adolescents’ sleep duration, adolescents’ physical activity, and adolescents’ consumption of vegetables, fruits, sugar-sweetened beverages, and meat.

When the relationship between maternal lifestyle and the risk of offspring MS components was investigated, the increased number of maternal ideal lifestyle factors was associated with decreased risk of abdominal obesity and low HDL-C in their offspring (*P* for trend <0.05). In addition, adolescents whose mother met 5–6 ideal lifestyle factors had lower risk of abdominal obesity (OR = 0.580, 95% *CI* = 0.375~0.897), elevated blood pressure (OR = 0.599, 95% *CI* = 0.390~0.918), and high triglycerides (OR = 0.627, 95% *CI* = 0.405~0.971), compared with those whose mother met 0–2 ideal lifestyle factors (**Table 5**).

**Table 5:** Associations between combined maternal lifestyle factors and risk of MS components in offspring.

Number of ideal lifestyle factor	MS components in offspring^†,‡^			
	Abdominal obesity	Elevated blood pressure	High triglycerides	Low HDL-C
0–2	1 (reference)	1 (reference)	1 (reference)	1 (reference)
3	0.726 (0.469, 1.122)	0.659 (0.429, 1.012)	0.651 (0.420, 1.010)	0.829 (0.504, 1.364)
4	0.662 (0.433, 1.010)	0.573 (0.378, 0.869)^*^	0.570 (0.372, 0.871)^*^	0.554 (0.340, 0.902)^*^
5–6	0.580 (0.375, 0.897)^*^	0.599 (0.390, 0.918)^*^	0.627 (0.405, 0.971)^*^	0.659 (0.399, 1.087)
*P* for trend	0.009	0.076	0.197	0.033

^*^ P < 0.05.

^†^In the regression model, we adjusted for region, adolescents’ age and sex, delivery way, feeding type, birth weight, parental education level, parental age, family history of diseases, paternal BMI, paternal cigarette smoking, paternal alcohol consumption, adolescents’ cigarette smoking, adolescents’ alcohol consumption, adolescents’ sleep duration, adolescents’ physical activity, and adolescents’ consumption of vegetables, fruits, sugar-sweetened beverages, and meat. ^‡^The results of high blood glucose were not shown because of few cases (n = 16).

^‡^The results of high blood glucose were not shown because of few cases (n = 16).

The association of maternal and offspring combined lifestyle with offspring MS risk was shown in **Table 6**. Compared with those had 0–3 ideal lifestyle factors in both mothers and offspring, ORs ranged from 0.818 (95% *CI* = 0.593~1.128) to 0.243 (95% *CI* = 0.169~0.349) in those with 4–6 ideal lifestyle factors in either mothers or offspring, and the MS risk was 75.7% lower (OR = 0.243, 95% *CI* = 0.169~0.349) in those had 4–6 ideal lifestyle factors in both mothers and offspring. Similar relationships were additionally observed when components of MS were estimated, the risk of abdominal obesity, elevated blood pressure, high triglycerides, low HDL-C were significantly lower in offspring had low-risk lifestyle in both mothers and themselves.

**Table 6:** Risk of offspring MS and its components according to the combination of maternal lifestyle and offspring lifestyle^†,‡^.

Maternal lifestyle	Offspring lifestyle	Abdominal obesity	Elevated blood pressure	High triglycerides	Low HDL-C	MS
High risk	High risk	1	1	1	1	1
Low risk	High risk	0.892 (0.704,1.129)	0.786 (0.614, 1.007)	0.922 (0.715, 1.188)	0.614 (0.466, 0.810)^*^	0.818 (0.593, 1.128)
High risk	Low risk	0.221 (0.160, 0.306)^*^	0.600 (0.447, 0.807)^*^	0.644 (0.474, 0.876)^*^	0.525 (0.374, 0.738)^*^	0.294 (0.182, 0.475)^*^
Low risk	Low risk	0.220 (0.172, 0.282)^*^	0.558 (0.439, 0.710)^*^	0.593 (0.462, 0.762)^*^	0.503 (0.384, 0.659)^*^	0.243 (0.169, 0.349)^*^

^*^P < 0.05.

^†^Adjusted for region, children’s age and sex, delivery way, feeding type, birth weight, parental education level, parental age, family history of diseases, paternal BMI, paternal smoking, and paternal drinking status.

^‡^The results of high blood glucose were not shown because of few cases (n = 16).

## Discussion

In this study, the lifestyle of mothers was strongly related with the risk of MS in their offspring, which was independent of offspring’s lifestyle. The more healthy lifestyle mothers adhered, the lower MS risk offspring had. In addition to maternal lifestyle, when both mothers and their offspring had more ideal lifestyle factors (4–6 ideal factors), the risk of offspring MS was lower than those whose mothers and themselves both had 0–3 ideal lifestyle factors.

Previous studies conducted in children and adolescents had shown that maternal weight, smoking, and alcohol consumption was related with offspring’s obesity, blood pressure, and triglycerides (11, 12, 27). Dhana and colleagues found that offspring of mothers who adhered to healthy lifestyle had a 73% lower risk of obesity (13). In the present study, we found a comprehensive maternal healthy lifestyle had a substantial protective effect on MS risk in offspring. Thus it can be seen, maternal lifestyle was not only associated with risk of offspring obesity, but also other cardio-metabolic factors, such as dyslipidemia and elevated blood pressure, which emphasized the importance of maternal lifestyle.

Because of the high consistency of lifestyle between mothers and offspring, it is wonder whether adolescents’ lifestyle could be a mediation between their health status and mothers’ lifestyle (28–30). Thus, the association was further adjusted for adolescents’ lifestyle factors. This adjustment further reduced the offspring MS risk. Though the mechanisms underlying were not elucidated, accumulated evidence has shown that parenting style and practices may contribute to the weak mediation effect of offspring’s lifestyle (31, 32). And in westernized context, there were differences regarding lifestyle perception and management in teenagers and their parents (33). In addition, adolescents of this age spent most of their time at school, and they were susceptible to the external environment beyond family (34), and the influence of peers become more influential during adolescence while the impact of mothers and other family members was attenuated (35). Furthermore, when mothers and offspring both had healthy lifestyle, the risk of MS in offspring was even lower. Our findings are in line with the study conducted in European children aged 5 to 13 years, which indicated that both parental and offspring’s healthy lifestyle committed to a healthier weight for children (30), and suggested a combined effect of maternal and offspring’s lifestyle on adolescents’ cardiometabolic health.

Moreover, the relationship of maternal lifestyle on adolescent MS risk was observed in different areas and sexes, it was more substantial in girls and those lived in urban area. These finding were also detected by another study conducted in Chinese families (36). In addition, studies have shown that parents living in urban region pay more attention to adolescents’ daily lifestyle (37). A stronger relationship between mothers and daughters had been detected, and this observation was in agreement with previous studies. The potential explanation for the different findings between boys and girls was that boys were more active and were more susceptible to the external environment than girls. In addition, the shared environmental components between mothers and daughters were stronger than those between mothers and sons (38), which may also relate to the epigenetic effects on intergenerational relationships between same sex (39). Hence, our findings suggested that adolescents would benefit from family-based intervention, especially in urban adolescents and girls.

Based on a comprehensive lifestyle, this study suggested that the development of mothers’ healthy lifestyle could provide further opportunity to improve offspring’s health, in addition to the improvement of adolescents’ lifestyle behavior. Our study highlights the critical role of maternal healthy lifestyle and could be useful to improve future intervention strategy to decrease the MS risk in youths. Intervening by focusing on lifestyle of mothers but preferably of both members of the mother-offspring dyad might be a novel and effective strategy for preventing MS.

Several limitations should be considered when our findings were interpreted. First, this study was a cross-sectional study and was unable to determine causal relationship between maternal lifestyle and risk of offspring MS. Further randomized controlled trial is needed to confirm our findings. In addition, the information of lifestyle was collected by self-reported questionnaire, objective and accurate measurements, such as accelerometers, are warranted. Moreover, our study only examined maternal lifestyle, and paternal overall lifestyle was not included, prospective study considering parental role in the development of MS in offspring is needed.

## Conclusion

The present study found that maternal healthy lifestyle was associated with decreased MS risk in offspring, which was independent of offspring’s lifestyle. What’s more, the adolescent MS risk was further lower when mothers and their offspring both follow a healthy lifestyle. These findings suggest the importance of maternal lifestyle on their offspring’s health and support the mother-based intervention strategy aimed to improve adolescents’ cardio-metabolic health.

## Data Availability Statement

The raw data supporting the conclusions of this article will be made available from the corresponding author upon request.

## Ethics Statement

The studies involving human participants were reviewed and approved by the Ethical Committee of the Peking University (Number: IRB0000105213034). Written informed consent to participate in this study was provided by the participants’ legal guardian/next of kin.

## Author Contributions

YL designed the study, carried out the data analysis, drafted the initial manuscript, and reviewed and revised the manuscript. ZY, XW, and DG carried out the initial data analysis, drafted the initial manuscript, and reviewed and revised the manuscript. ZZ collected data, critically reviewed the manuscript, and contributed to the interpretation of results. LA polished the language and reviewed and revised the manuscript. JM and BD conceptualized and designed the study, coordinated and supervised the data collection, and critically reviewed the manuscript for important intellectual content. All authors contributed to the article and approved the submitted version.

## Funding

This study was supported by the Research Special Fund for Public Welfare of Health (No. 201202010) and National Natural Science Foundation of China (No. 81903344) and Excellent Talents Fund Program of Peking University Health Science Center (BMU2017YJ002).

## Conflict of Interest

The authors declare that the research was conducted in the absence of any commercial or financial relationships that could be construed as a potential conflict of interest.
